# A new postural stability-indicator to predict the level of fear of falling in Parkinson’s disease patients

**DOI:** 10.1186/s12938-020-00808-w

**Published:** 2020-08-18

**Authors:** Ehsan Pourghayoomi, Saeed Behzadipour, Mehdi Ramezani, Mohammad Taghi Joghataei, Gholam Ali Shahidi

**Affiliations:** 1 Department of Neuroscience, Faculty of Advanced Technologies in Medicine, Iran University of Medical Sciences, Tehran, Iran; 2 Mechanical Engineering Department, and Cross Appointed with Djawad Movafaghian Research Center in Neuro-rehabilitation Technologies, Sharif University of Technology, Tehran, Iran; 3 Cellular and Molecular Research Center, Iran University of Medical Sciences, Tehran, Iran; 4 Movement Disorders Clinic, Hazrat Rasool Hospital, Iran University of Medical Sciences, Tehran, Iran

**Keywords:** Fear of falling, Parkinson’s disease, Postural control, Force platform, Diagnosis

## Abstract

**Background:**

Fear of falling (FoF) is defined as a lasting concern about falling that causes a person to limit or even stop the daily activities that he/she is capable of. Seventy percent of Parkinson’s disease (PD) patients report activity limitations due to FoF. Timely identification of FoF is critical to prevent its additional adverse effects on the quality of life. Self-report questionnaires are commonly used to evaluate the FoF, which may be prone to human error.

**Objectives:**

In this study, we attempted to identify a new postural stability-indicator to objectively predict the intensity of FoF and its related behavior(s) in PD patients.

**Methods:**

Thirty-eight PD patients participated in the study (mean age, 61.2 years), among whom 10 (26.32%) were identified with low FoF and the rest (73.68%) with high FoF, based on Falls Efficacy Scale-International (FES-I). We used a limit of stability task calibrated to each individual and investigated the postural strategies to predict the intensity of FoF. New parameters (*FTR*_*i*_s; functional time ratio) were extracted based on the center of pressure presence pattern in different rectangular areas (*i* = 1, 2, and 3). The task was performed on two heights to investigate FoF-related behavior(s).

**Results:**

*FTR*_*1*/2_ (the ratio between *FTR*_*1*_ and *FTR*_*2*_) was strongly correlated with the FES-I (*r *= − 0.63, *p *< 0.001), Pull test (*r* = − 0.65, *p *< 0.001), Timed Up and Go test (*r *= − 0.57, *p* < 0.001), and Berg Balance Scale (*r *= 0.62, *p* < 0.001). The model of *FTR*_*1*/2_ was identified as a best-fitting model to predicting the intensity of FoF in PD participants (sensitivity = 96.43%, specificity = 80%), using a threshold level of ≤ 2.83.

**Conclusions:**

Using the proposed assessment technique, we can accurately predict the intensity of FoF in PD patients. Also, the *FTR*_*1/2*_ index can be potentially considered as a mechanical biomarker to sense the FoF-related postural instability in PD patients.

## Background

Fear of falling (FoF) is common in the elderly, particularly in most Parkinson’s disease (PD) patients [1]. It is known as a risk factor for recurrent falls in PD patients [1–3]. FoF is considered as a lasting concern about falling that causes a person to limit or even stop the daily activities that he/she can do [4, 5]. FoF is protective when it interferes with dangerous activities [4], and may even be useful in preventing falls [6]. On the other hand, it can be maladaptive and restrict patients in their daily activities [6]. Previous studies have shown a high correlation between FoF and reduced life-space mobility [4, 5]. Ultimately, FoF leads to deconditioning, functional decline, and reduced quality of life [5, 7]. Seventy percent of PD patients reported activity limitations due to FoF [8]. Therefore, the timely and accurate diagnosis of FoF in PD patients is critical to prevent its additional harmful effects.

Previous studies have widely used the Falls Efficacy Scale-International (FES-I) as a valid and reliable subjective questionnaire to assess FoF in community-dwelling populations [9, 10]. Among the FoF evaluation questionnaires, the FES-I (*ICC* > 0.9) has been suggested as an adequate scale to evaluate the FoF in the PD population [11]. The FES-I, however, is a self-report questionnaire, and its outcome may be biased [12, 13].

Postural instability in the PD is known as an independent risk factor for restricting mobility [14] and increasing FoF [15]. In this regard, previous studies showed that FoF questionnaires are significantly correlated with some basic postural stability measures [16–18]. Pull test (PT), Berg Balance Scale (BBS), and Timed Up and Go (TUG) are commonly used to assess postural stability in PD patients [14]. There are some limitations to them, for instance, the ceiling effects of the BBS, which indicate it can be misleading during the evaluation of patients with mild deficits [14]. Furthermore, the PT involves external perturbations that, to a large extent, depend on the examiner’s skills to running the test and interpreting results [19]. Overall, basic clinical measures only provide a gross indicator of postural control efficiency [20].

Postural control instrumented-tests provide unbiased measurement and detailed analysis of postural control performance and associated strategies [20]. These kinds of measures are of interest to researchers and clinicians for the accurate identification of insufficient postural stability in PD patients [21]. A significant association between FoF and postural control, which is assessed by the center of pressure (CoP) measures [22, 23], has been reported. The CoP data have usually been used to assess body sway in static [24, 25] and dynamic [26, 27] situations. Studies have shown that the quiet standing position (static task) may be unable to demonstrate postural control deficiencies [28]. On the other hand, dynamic standing posture tasks are suggested to consider in FoF studies [29]. The limit of stability (LoS) was used as a dynamic standing posture task [27, 30]. In a LoS task, the subject attempts to move his CoP from the stability region away in different directions, without losing his balance and taking a step [31]. Many studies investigated postural stability using laboratory LoS tasks in PD [15, 27, 32, 33]; however, to the best of our knowledge, there is no evidence to show their relationship with FoF in these patients. Some studies, for example, showed that PD patients underperformed in the mediolateral [27, 32] and posterior [32] body excursion compared to healthy elderly subjects. Other studies have also confirmed the existence of insufficient postural stability in the anterior, posterior [15], forward-right, and backward-left [33] directions in the PD population. It seems that PD patients show postural instability, approximately, in all directions while performing a LoS task. Also, in addition to the dominant side, unilateral involvement in the early stages of PD [33] can affect the results of particular directions in a LoS task. As a result, a multi-directional approach is required to investigate FoF by a LoS task.

Inspired by the mentioned evidence, we aimed to introduce a new postural stability-indicator that can objectively predict the intensity of FoF in PD patients. We hypothesized, based on the intensity of FoF, there is a different postural strategy in the individuals’ area of abilities. Therefore, we used a LoS task calibrated to each individual and investigated the postural strategies to predict the intensity of FoF. We established a new perspective to analyze CoP data and investigated the pattern of CoP presence in different areas as a multi-directional approach. The proposed tool can help clinicians to more accurately identify the level of FoF in PD patients. Accurate prediction leads to timely intervention, such as rehabilitation protocols (e.g., exercise [34] and cognitive behavioral therapy [35]), to maintain and improve the quality of life in PD patients.

## Results

Independent sample *t* test showed no significant difference between low-FoF and high-FoF groups for the age (*p* = 0.06), height (*p* = 0.12), weight (*p* = 0.06), cognitive performance (*p* = 0.08), and psychological distress (*p* = 0.06). These results confirmed the homogeneity between two groups. The mean and standard deviation for outcomes of the clinical assessments are shown in Table 1.

**Table 1:** Mean and standard deviation of the demographic characteristics and clinical assessments

	Total (*N *= 38)	Low-FoF group (*n *= 10)	High-FoF group (*n *= 28)	*p*-*value*
Age (years)	60.76 (9.39)	56 (8.71)	62.46 (9.18)	0.06
Height (cm)	171.39 (5.13)	173.55 (4.12)	170.62 (5.30)	0.12
Weight (kg)	77.52 (13.12)	84.1 (8.64)	75.17 (13.75)	0.06
MoCA	21.78 (4.64)	24 (3.02)	20.96 (4.9)	0.08
HADS-total	14.88 (6.66)	11.44 (7.26)	16.1 (6.1)	0.06
HADS-anxiety	7.91 (4.03)	6.44 (4.92)	8.44 (3.62)	0.18
HADS-depression	6.97 (3.62)	5 (3.23)	7.67 (3.55)	0.04*
FES-I	29.13 (10.25)	17.9 (2.08)	33.14 (8.89)	< 0.001**
Duration of disease (years)	7.36 (4.98)	4.2 (1.71)	8.51 (5.3)	0.02*
HY stage	2.36 (0.68)	1.6 (0.52)	2.63 (0.5)	< 0.001**
PT	1.18 (0.93)	0.3 (0.48)	1.5 (0.79)	< 0.001**
BBS	46.84 (5.86)	53.3 (2.41)	44.5 (4.91)	< 0.001**
TUG	8.01 (1.65)	6.63 (0.73)	8.5 (1.61)	0.001**

*BBS* Berg Balance Scale, *FES*-*I* Falls Self-Efficacy Scale-International, *FoF* Fear of Falling, *HADS* Hospital Anxiety and Depression Scale, *HY* Hoehn and Yahr, *MoCA* Montreal Cognitive Assessment, *PT* Pull Test, *TUG* Timed Up and Go

**p-value < .01; *p-value < .05

### FTRs’ reliability

*FTR*_*1*_ and *FTR*_*2*_ were found to have high relative (*ICC* ≥ 0.75) and absolute (%*SEM* ≤ 10%) reliability at all conditions (levels of height) (Table 2).

**Table 2:** The intra-class correlation (*ICC*), and the percentile standard error of measurement (%*SEM*) of *FTRs*

Level of height	*FTR*_*1*_	*FTR*_*2*_	*FTR*_*3*_
Ground			
*ICC*_(2,3)_	0.84	0.89	0.68
*SEM* (%*SEM*)	2.8 (4.9%)	2.5 (8.8%)	1.9 (12.78%)
20 cm			
*ICC*_(2,3)_	0.89	0.88	0.79
*SEM* (%SEM)	2.7 (4.59%)	2.6 (9.46%)	1.62 (11.93%)
40 cm			
*ICC*_(2,3)_	0.91	0.93	0.76
*SEM* (%SEM)	2.35 (3.88%)	1.91 (7.04%)	1.65 (13.48%)

*FTR* Functional Time Ratio

### Correlation analysis

The correlation between the CoP data and the main score of basic clinical measures was investigated (Table 3). *FTR*_*1*_ and *FTR*_*2*_ were strongly correlated with the basic clinical measures (*p* < 0.001), whereas there was a negligible/weak correlation between the *FTR*_*3*_ and these measures (*p* > 0.05). Therefore, convergent validity for *FTR*_*1*_ and *FTR*_*2*_ was established to estimate the level of FoF (Table 3).

**Table 3:** The correlation coefficient (*R*^*2*^) between CoP parameters and basic clinical measures

	FES-I	PT	BBS	TUG
*FTR*_*1*_	−0.630* (0.396)	−0.661* (0.476)	0.621* (0.385)	−0.614* (0.377)
*FTR*_*2*_	0.668* (0.446)	0.634* (0.404)	−0.596* (0.355)	0.615* (0.378)
*FTR*_*3*_	−0.087 (0.007)	0.209 (0.041)	−0.107 (0.012)	0.028 (0.001)
*FTR*_*1/2*_	−0.630* (0.397)	−0.653* (0.403)	0.618* (0.382)	−0.574* (0.329)

*BBS* Berg Balance Scale, *FES*-*I* Falls Self-Efficacy Scale-International, *FTR* Functional Time Ratio, *PD* Parkinson Disease, *PT* Pull Test, *TUG* Timed Up and Go

*Correlation is significant at the 0.01 level

### The effect of threatening conditions on postural strategies

**Fig. 1:**
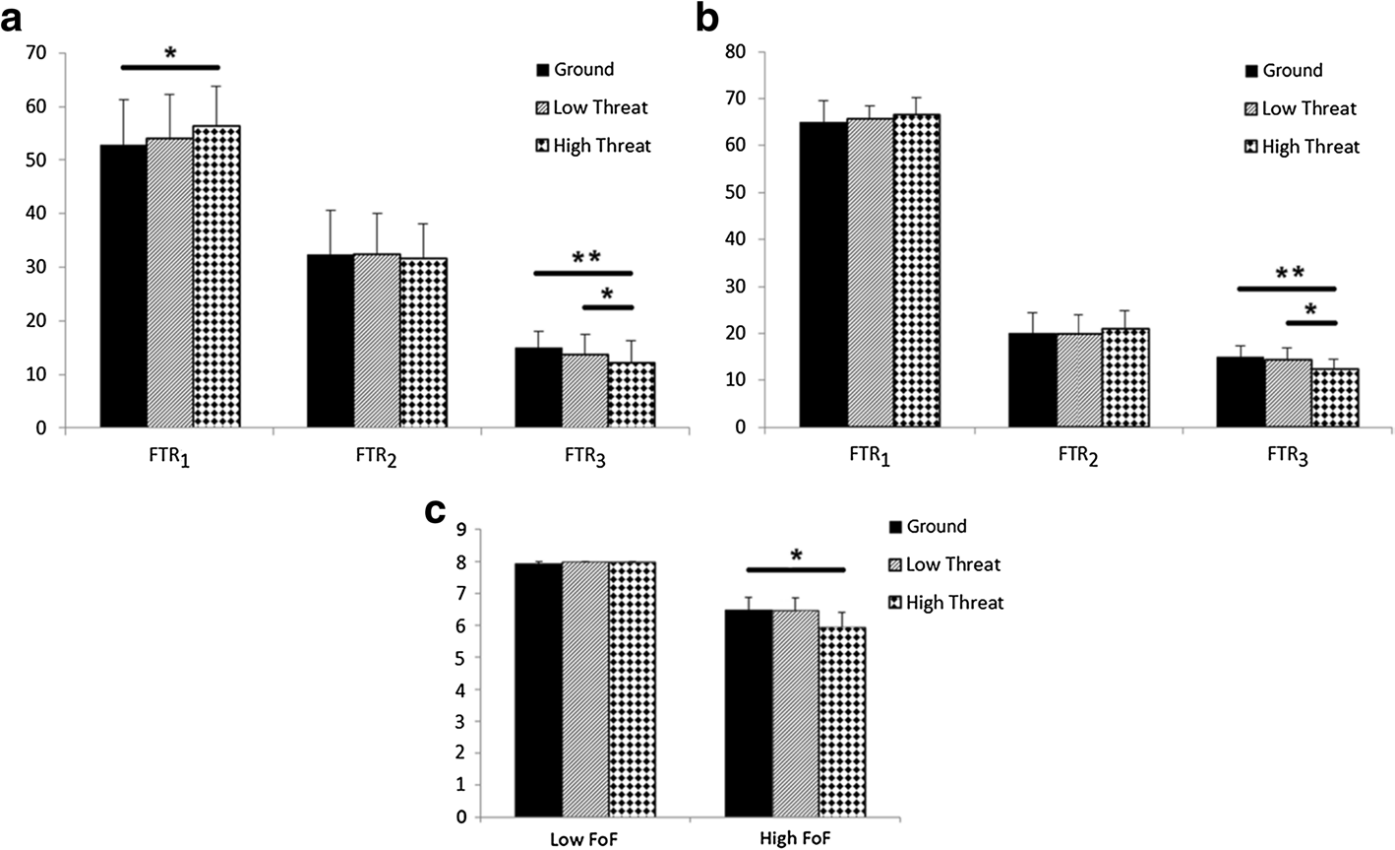
Trends of change of FTRs and total score in the LoS task in the face of threatening conditions. **a** FTRs in the high-FoF participants; **b** FTRs in the low-FoF participants; **c** total score of LoS in two groups. The significance of the differences is shown by bold line and stars (** *p*-value < .01, * *p*-value < .05). *FTR* Functional Time Ratio, *FoF* Fear of Falling

The repeated-measure ANOVA analysis for the high-FoF group showed significant difference (*p* < 0.01) for the *FTR*_*1*_ and *FTR*_*3*_. Bonferroni post hoc results showed that these differences existed between the ground and 40-cm levels (*FTR*_*1*_, *p* = 0.015; *FTR*_*3*_, *p* = 0.001) and between the 20-cm and 40-cm levels (*FTR*_*3*_, *p* = 0.027) (Fig. 1a). In the low-FoF group, the repeated-measure ANOVA showed a significant difference (*p* = 0.001) only for *FTR*_*3*_. Bonferroni post hoc results indicated that the difference existed between the ground and 40-cm levels (*p* = 0.002) and between 20-cm and 40-cm levels (*p* = 0.011) (Fig. 1b).

The Freidman test for the high-FoF group showed a significant difference for the total score of LoS, *X*^*2*^ (2) = 11.58, *p* = 0.003. Dunn–Bonferroni post hoc tests were carried out and there was a significant difference between ground level and 40-cm level, *p* = 0.012 (Fig. 1c).

### Predicting the level of FoF

A very high correlation was obtained between *FTR*_*1*_ and *FTR*_*2*_ (*ICC* = −0.948, *p* < 0.001). Since the lack of multi-collinearity between variables is a precondition for the logistic regression analysis [13], and in anticipation of the level of FoF, the *FTR*_*1*_ and *FTR*_*2*_ had the opposite direction (Table 3), we introduced the new parameter, *FTR*_*1/2*_, which is the ratio between the *FTR*_*1*_ and *FTR*_*2*_.

Overall, four binary logistic regression models were built to predict the odds of a participant being in the low or high-FoF groups based on the *FTR*_*1*_, *FTR*_*2*_, *FTR*_*1/2*_-ground, and *FTR*_*1/2*_-40 cm, separately. As shown in Table 4, all models worked well in distinguishing each participant’s group. ROC curve analysis confirmed that the *FTR*_*1*_, *FTR*_*2*_, and *FTR*_*1/2*_ had significant power to discriminate low-FoF and high-FoF groups (Table 4). As a result, *FTR*_*1/2*_ at ground level, with the lowest *CAIC*, was the best-fitting model for predicting the intensity of FoF, using a threshold level of ≤ 2.83. Also, *FTR*_*1/2*_ can consistently differentiate low-FoF and high-FoF individuals with low classification error (Table 4c).

**Table 4:** Binary logistic regression models and their evaluations

(a) Binary logistic regression outcomes based on *FTR*_*1*_,*FTR*_*2*_, and *FTR*_*1/2*_ parameters				
Models	Odds ratio	*p*-value	95% *CI* for odds ratio	
			Lower	Upper
*FTR*_*1*_	1.54	0.005^*^	1.14	2.07
*FTR*_*2*_	1.65	0.01^*^	1.13	2.42
*FTR*_*1*/2_-ground	33.55	0.005^*^	2.91	386.72
*FTR*_*1*/2_-40 cm	30.55	0.005^*^	2.78	335.73

(b) Receiver operating characteristic (ROC) curve analysis and corrected Akaike’s Information Criterion (*CAIC*) of *FTR*_*1*_, *FTR*_*2*_, and *FTR*_*1/2*_				
	*FTR*_*1*_	*FTR*_*2*_	*FTR*_*1/2*_-ground	*FTR*_*1/2*_-40 cm
*AUC*	0.921	0.932	0.939	0.921
95% *CI* for *AUC*	0.830 to 1	0.845 to 1	0.860 to 1	0.835 to 1
*p*-value	< 0.0001^**^	< 0.0001^**^	< 0.0001^**^	< 0.0001^**^
Overall accuracy	86.84	89.47	92.1	89.5
Sensitivity	89.29	92.9	96.43	96.43
Specificity	90	80	80	70
*CAIC*	24.1	22.58	21.56	22.9
Optimal cutoff value	≤ 59.43	> 21.36	≤ 2.83	≤ 2.71
(c) Cross-validation results				
Error	15.79	21.05	10.53	13.16
Sensitivity	82.14	71.43	92.86	92.86
Specificity	90	88.57	80	70

*AUC* Area Under the Curve, *CI* Confidence Interval, *FTR* Functional Time Ratio

**p-value ≤ .0005; *p-value < .05

## Discussion

FoF is defined as a lasting concern about falling that causes a person to limit or even stop the daily activities that he/she is capable of [4, 5]. Inspired by this definition, we used LoS, a postural task in the area of individual abilities, and the postural control strategies to objectively identify FoF. We calibrated the LoS task based on 75% of each participant’s ability. Using the LoS task, we introduced a new postural stability-indicator to predict the level of FoF in PD patients. In this regard, with a new method for analyzing the CoP data, three indices, *FTR*_*1*_, *FTR*_*2*_, and *FTR*_*3*_ were introduced. Ultimately, the ratio between *FTR*_*1*_ and *FTR*_*2*_ *(FTR*_*1/2*_) showed a 92.1% overall accuracy to predict the level of FoF in participants.

### FTRs’ reliability

The reliability of *FTR*_*1*_, *FTR*_*2*_, and *FTR*_*3*_ was tested with an ANOVA-based *ICC* model. Three different test conditions were used (levels of height). Our results showed that *FTR*_*1*_ and *FTR*_*2*_ had a high relative and absolute reliability in all conditions. Also, acceptable to high relative reliability and acceptable absolute reliability were obtained for *FTR*_*3*_. These results showed intra-session reliability for our measures. We, therefore, recommend researchers to select these measures in future research and assessment.

### Correlation analysis

Kumar et al. showed a significant correlation between the FoF and functional balance (–0.97, *p* < 0.01) and mobility (0.95, *p* < 0.05) measures in the elderly population [17]. Also, some studies in the PD population [16, 18] showed a correlation between them. PT and TUG had negative correlations with postural stability and BBS had a positive correlation with postural stability [14]. Therefore, it can be concluded the low-FoF group has better postural stability than the high-FoF one. It is in agreement with previous studies, which confirmed a significant correlation between FoF and postural stability [15, 22, 23].

According to our results, *FTR*_*1*_ and *FTR*_*2*_ were strongly correlated with clinical postural stability measures. Also, *FTR*_*1*_ and *FTR*_*2,*_ respectively, had a negative and positive correlation with FES-I. Our results confirmed that participants with a prolonged presence of CoP in the *RFA*_*1*_ had better postural stability and lower FoF than those with a prolonged presence of CoP in the *RFA*_*2*_. It seems that patients with high FoF probably had lower accuracy in controlling and guiding their CoP toward targets and returning to the home position, due to insufficient postural stability; therefore, they spend more time in *RFA*_*2*_. In contrast, participants with low FoF probably had higher performance in controlling the CoP motion (sufficient postural stability). Therefore, they could quickly hit the targets and returned to the home position, and spent more time in *RFA*_*1*_. These results are supported by a previous study [32], which demonstrated that the directional control is poorer for PD patients in comparison to the healthy population. Regarding the HY stages being positively correlated with FoF (*r*_*s*_ = 0.47, *p* < 0.001) [16], a possible reason for this similarity is that their participants [32], similar to high-FoF participants in our study (mean ± SD of HY = 2.63 ± 0.5), were in HY stages of 2 to 3. It is necessary to mention that the PD duration increases from HY Stage 1 to Stage 5 [36]. Moreover, Lindholm et al. [8] showed a significant correlation between PD duration and FoF (0.35, *p* < 0.001). In line with these evidences, in our study, the PD duration of high-FoF group was significantly higher than low-FoF group. Overall, it seems that, in advanced stages of PD, because of higher FoF, participants had poorer performance in the LoS task.

### The effects of the threatening conditions on postural strategies

Previous studies investigated the behavioral correlations of FoF with increased heights [37]; therefore, we ran the LoS task at two other elevated levels to investigate preferred postural strategies in patients with low and high FoF.

It seems that participants in both groups modified their postural strategies in the high threat conditions (Fig. 1). According to the results of the repeated-measure ANOVA, the low-FoF participants significantly decreased their presence time in *RFA*_*3*_ and spent more time in other areas (Fig. 1b). High-FoF participants, in addition to decreased presence time in *RFA*_*3*_ and the total score of LoS task, significantly increased their presence time in the *RFA*_*1*_ (Fig. 1a, c). It indicates that in the challenging conditions, subjects of the high-FoF group attempt to reduce the peripheral mobility of the CoP and increase the presence time at home position. These results comply with the reduced spatial mobility in the FoF population, reported by previous studies [4, 5]. Jefferis et al. [5] showed that elderly men with FoF had lower excursions from the home position and more mobility difficulties than those without FoF. This observation shows that our perspective is in line with the concept of FoF.

Adkin et al. [38] mentioned that the central nervous system (CNS) progressively tightens the control of posture when the postural threat increases. Their study, accompanied by other research works [39, 40], well established that the CoP sway amplitude significantly decreases with increasing level of threat. In the present study, we ran the LoS task on different heights in an ascending order, which corresponds to the level of the threat. Therefore, the mentioned CNS changes in our participants are also expected. Some studies revealed the underlying neuromuscular strategy [38, 41]. They suggest that after increasing levels of threat, the CNS applies a ‘stiffening strategy’ [29, 38, 41] that leads to reducing the degree of freedom. In this strategy, reflexive muscle co-contractions occur around the ankle joint to maintain the body in the desired position in response to the threat [18, 42, 43]. In our study, participants were asked to use an ankle strategy rather than a hip strategy. It is possible that due to the effects of stiffening strategies around the ankle joint in threatening conditions, participants in the low and high-FoF groups switched to a hip strategy for performing the LoS task. Therefore, we suggest future studies to identify the postural segmental strategies and the neuro-muscular pattern underlying this behavior by kinematic and electromyography devices.

One possible reason for inefficient postural adjustment to perform the task is poor movement planning [29]. In a study on older adults with low and high FoF, participants in both groups showed an initial attentional bias toward fall-threatening words, compared to threatening words unrelated to falling [44]. Zaback et al. [45] also reported the attention shifted to movement processes, threat stimuli, and self-regulation in a postural-threat condition. They concluded that these attention shifts are associated with changes in postural control [45]. On the other hand, many researchers have interpreted stiffening strategies as an intuitive preparatory strategy for accommodating potential destabilizing situations [29, 38, 41, 46]. Based on the described evidences, we expected that in threatening conditions, a stiffening strategy was automatically developed in both groups of PD with low and high FoF. Regarding *FTR*_*1/2*_ and the total score of LoS (Fig. 1), it seems that CNS, based on the intensity of FoF, showed a different adaptation with a stiffening strategy. Participants with high FoF probably were unable to overcome the stiffening strategy, and it led to restricting themselves to complete the LoS task (hitting the targets) (Fig. 1c). Attentional control theory predicts that anxious people, due to failure in shifting attention from task-irrelevant toward task-relevant information, are unable to properly plan the movement [29]. Based on independent *t* test for MoCA and HADS-anxiety subscale (*p* = 0.08, *p* = 0.18, respectively), It seems that the cause for group differences in movement planning was unrelated to general cognitive or anxiety levels. According to the HADS-depression subscale (*p* = 0.04), scores of the high-FoF participants were significantly higher than the low-FoF ones. A study [47] reported that depression disorder is associated with neurocognitive changes related to coordinate motor output. Therefore, depressive symptoms may be the cause of improper motor planning in PD patients with high FoF. In line with this perception, Franzén et al. [48], for the first time, showed depressive symptoms as the strongest independent variable (*β* = 0.40, *p* < 0.001) to predict concerns about falling in PD. Depression is closely associated with PD [49, 50]. The development of depression in PD is more likely to be caused by the nigrostriatal pathway degeneration than the outcome of the awareness of the disease’s prognosis [51]. Motor symptoms of PD emerge when 50% of dopaminergic neurons degenerate, while depressive symptoms are prevalent even before the onset of motor symptoms [51, 52]. Some of the factors that consistently correlate with depression in PD include earlier-onset, advanced stage, psychiatric comorbidity (e.g., anxiety), and the presence of cognitive decline [53]. It seems that by managing depression in the PD population, we can prevent additional syndrome, such as fear of falling. Cognitive–behavioral therapy showed satisfactory effects to control the FoF [54] and depression [52, 54–56] in PD, which needs further study. Furthermore, we suggest that researchers consider the level of depression while investigating the mechanism of FoF in participants with PD.

In the present study, for the first time, we investigated the behavior of PD patients in the LoS task in presence of height threat. Increasing the height should be above some threshold to impact the *FTR*s. There were no significant changes in the *FTR*s from the ground to the 20 cm height (Fig. 1). Another study has considered 19 cm as a low-threat condition [23], which is in line with our findings. Although the highest threat level (40 cm height) in this study was lower than those reported in the previous studies (140 cm, 160 cm and 320 cm) [37, 57], it left a significant effect on postural stability in the PD patients. Possible reasons are the multi-directional and dynamic nature of the LoS task and the simultaneous three-directional threat (setting the balance board on the front and side edges of the wooden platforms); whereas, tasks in the previous studies often were done in the quiet stance, away from the edge or with a uni-directional threat [37, 57]. In agreement with our study, Yiou et al. [26] showed that simultaneous multi-direction threats affect postural stability in dynamic tasks even in lower heights. Another rationale for using these heights was to identify the lowest heights that could significantly change the postural stability in PD patients. This finding simplifies the future studies for performing a dynamic task in PD patients at a high threat elevation. For future studies, we suggest that researchers investigate the impact of height threat on postural stability for other pathological diseases or the elderly population.

### Predicting the level of FoF

We demonstrated that by the proposed protocol on the ground level and *FTR*_*1*/2_ index, it is possible to predict the FoF level in PD subjects with an overall accuracy of 92.1% (Table 4). As mentioned above, the sufficiency of postural stability has a strong negative correlation with FoF. However, participants 3 and 6 (P3 and P6) of the low-FoF group, and participant 26 (P26) of the high-FoF group, based on *FTR*_*1/2*_, exhibited different behavioral strategies from their groups and fell in the opposite group. P3 and P6 had HY stages from 1 to 1.5 and had high postural stability, based on the objective postural stability measures (BBS score = 53 and 55, TUG = 7.07 and 5.97, and PT = 0). P26 was in HY stage of 3 and had postural instability and fall risk [58] based on clinical evidence (BBS = 43, TUG = 7.53 and PT = 1). As mentioned in methods, before LoS evaluation, participants learned the LoS task (6 repetitions). A previous study [59], confirmed the importance of repetition in the adaptation of emotional states. Therefore, P26 performed the LoS task in his secure LoS area, with sufficient postural stability similar to the low-FoF participants. To confirm this result, using high threat conditions (40 cm height), without pre-training at this height, we stimulated FoF in participants. Based on the different intensity of FoF in two groups, we expected different strategies, which were confirmed in the previous section. As we expected, based on *FTR*_*1/2*_-*40* *cm*, P26 was placed in the high-FoF group (his report in FES-I). P6 was still identified as high FoF. He was available for a follow-up, and his FES-I score obtained 8 months later, which indicated a sharp increase from 18 (baseline test) to 27. This suggests that his response to FES-I might have been biased at the baseline test or he had been developing FoF identified by *FTR*_*1/2*_. In other words, it seems that maladaptive high FoF might have developed in the early stages of disease, before the clinical diagnosis of the postural instability. Others have also reported that PD influences the movement preparation phase, even before the clinical detection of postural instability [60]; The cause has been reported to be an injury to the basal ganglia, which leads to the loss of automatic selection and execution of motor plans [61, 62]. Therefore, The *FTR*_*1/2*_ index seems to have the potential to be a mechanical biomarker to sense FoF-related postural instability.

This work had some limitations which should be considered in the interpretations of the results: off-drug state and female PD subjects were not included in this study.

## Conclusions

In this study, using a LoS task and based on a new perspective in analyzing CoP data, we identified a new set of postural stability indexes to predict the level of FoF in PD patients. The results confirmed the reliability and validity of the proposed indexes. Since new indexes are shown to be highly correlated with BBS, which assess the risk of falling, they have potential to be used as a screening tool for the risk of fall. The logistic model with *FTR*_*1/2*_ at the ground level was found to be the best-fitting one to predict our participants in the low or high-FoF groups (sensitivity = 96.43%, specificity = 80%). We also found the 40 cm height as the lowest level that has an impact on the behavior of PD participants in a LoS task. This study also reiterated the importance of managing depression of PD patients in FoF interventions and investigations. Ultimately, using timely interventions, we can help these patients preventing additional harmful effects of FoF (such as risk of falling) and improve their quality of life.

## Methods

### Participants

Thirty-eight PD patients were selected by a convenient sampling method from hospitals and rehabilitation centers affiliated with the Iran University of Medical Sciences, Tehran, Iran. The minimum sample size^1^ was determined based on the work of Peduzzi et al. [63]. Only male subjects were selected to avoid potential gender differences in functional mobility tasks [64]. Participants were divided into two groups of low FoF and high FoF, using the cut-off point level of the FES-I questionnaire (Table 1) [65]. An experienced neurologist diagnosed subjects with idiopathic PD and confirmed the stage of disease based on the Hoehn and Yahr (HY) scale [66]. In this study, patients were in HY stages 1 to 3. They were able to stand independently for at least 10 min. Participants with anxiety disorders and anti-anxiety drug consumption were excluded. We also omitted patients with severe dyskinesia and other neurological disorders (e.g., stroke). Patients with disease-related surgeries (e.g., deep brain stimulation), severe musculoskeletal impairment, and any other debilitating conditions were also excluded. Before taking part in this study, participants were fully informed of the experimental protocol, and the written consent, approved by the local ethics committee, was obtained.

### Balance board and designing the LoS task

**Fig. 2:**
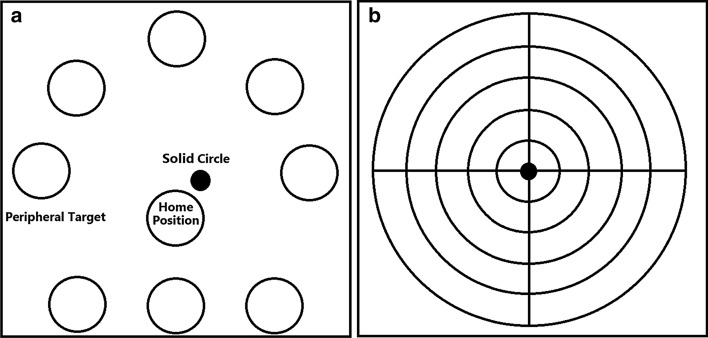
The graphical interface of the tasks. **a** The LoS task, with the home position in the middle, the real-time position of the CoP was shown as a solid circle, and 8 targets located around the home position; **b** the interface for finding the home position, the range of movement in the four directions, and the corresponding calibration of the position of the targets

We utilized a computerized system to implement the LoS task. It consisted of a PC and a monitor to run the graphical interface and provide the visual feedback for the participants, a CoP sensor called balance board (30.5 cm* 49.5 cm* 5.5 cm), and the required software. The balance board used four strain gauge load cells and a customized high-precision (24-bit) analog-to-digital converter to measure and transmits the position of the CoP to the PC at a sampling frequency of 100 Hz. We used custom software to interface the balance board with the computer. The software provided real-time feedback of the CoP position on a LCD monitor and guided the participant through the LoS task. The LoS task consisted of eight targets distributed around a circle and included a target at the center as the home position (Fig. 2a). Targets started to blink in random order. The participant moved his CoP, which was displayed on the same screen as a solid circle, to hit the blinking target. We considered 10 s to hit each target. After hitting each target, the central one blinked, and the participant returned to the home position. He had to remain in the home position for 5 s, and then the next target blinked.

The home position was determined by averaging the CoP position during the first 5 s in the quiet standing position. The distance of targets to the home position was calibrated individually for each participant. This step was performed before starting the LoS task. In the calibration step, the participant was asked to move his CoP as much as possible in the forward, backward, medial, and lateral directions (Fig. 2b). Three attempts were recorded, and 75% of the maximal excursion of CoP for each direction was considered as a secure distance between the targets and the home position. In the LoS task, participants were asked to use an ankle strategy rather than a hip one. They were also instructed to move their CoP as quickly and as accurately as possible from the home position to the peripheral targets and vice versa (Fig. 2a).

### Measurements and procedures

The demographic data, the level of general cognitive health (Montreal Cognitive Assessment, MoCA questionnaire), and the psychological distress (Hospital Anxiety and Depression Scale, HADS questionnaire), were collected (Table 1). Then, participants took clinical (FES-I, PT, BBS, and TUG) and LoS tests. The tests were administered before noon, approximately 1.5 to 2 h after their first drug intake (on-state phase).

In the FES-I, participants recorded the degree of their concern about falling in 16 activities of daily life on a scale of 1 to 4 [9]. The total score ranges from 16 (a minimum score which reflects no concern about falling) to 64 (a maximum score which reflects severe concern about falling). Based on the cutoff point level defined by Delbaere et al. [65], patients were classified into two groups: low FoF (total score of 16–22) and high FoF (total score of 23–64). The PT is a part of the Unified Parkinson Disease Rating Scale, and often has been used to identify postural instability. To perform PT, in standing position, the examiner pulls the subject backward at the shoulders and grades the response. This test scored from zero (normal) to 4 (unable to stand without assistance) [14]. The BBS is a 14-item scale to assess the balance in PD patients, mostly in mild to moderate disease stages. Score of each task ranges from zero to 4, and the maximum score is 56, which indicates perfect performance [14]. In the TUG test, the subject is asked to rise from a chair, walk a distance of 3 m, coming back, and sit down on the chair. The participant should be walking at maximum speed. The time (seconds) was recorded at three separate trials, and the mean was used [24].

**Fig. 3:**
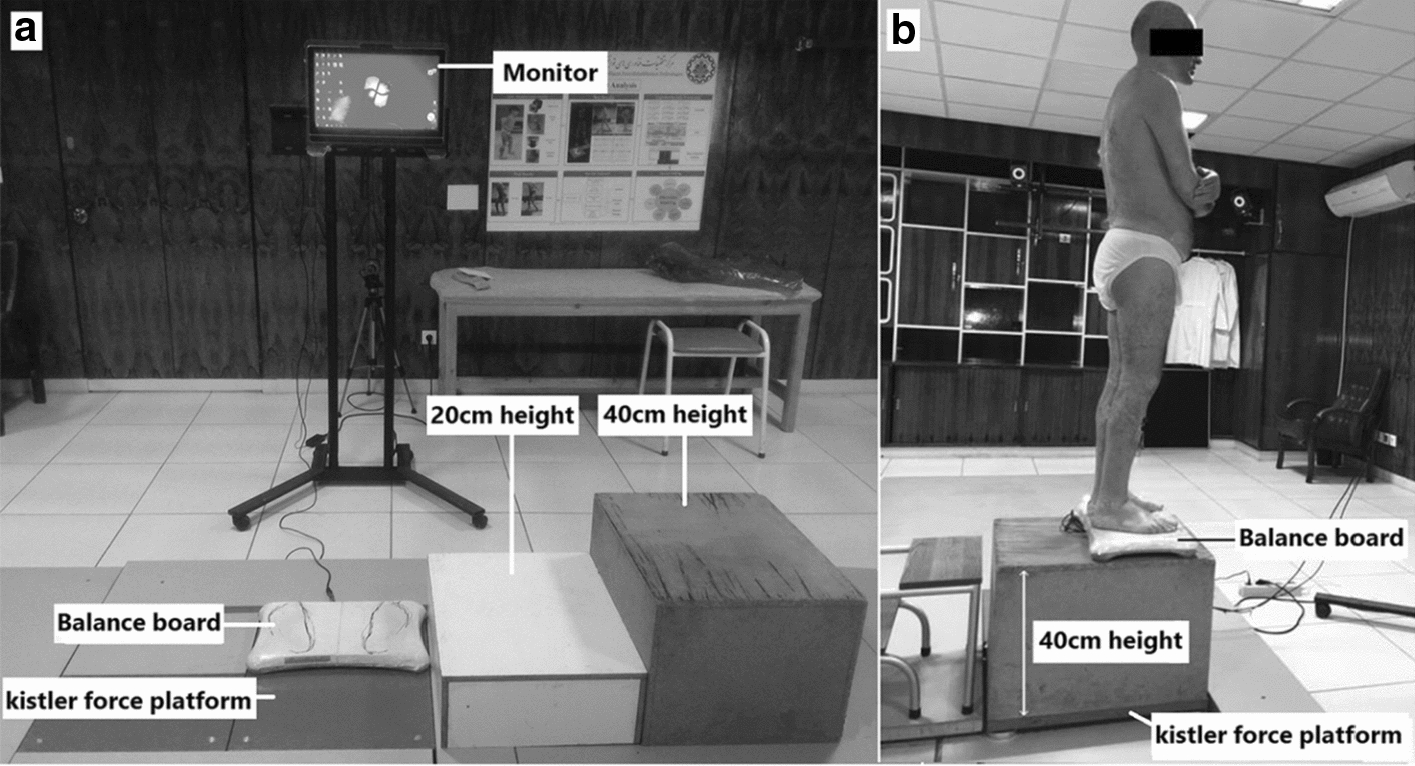
The experimental setup: **a** Instrument; **b** a participant taking the test

The Kistler force plate (60 cm*50 cm*7.5 cm_model: 9260AA6, version: 5.3.0.7, Switzerland), with a sampling rate of 1200 Hz, was used for accurate CoP recording (a customized balance board was used to provide visual feedback from the CoP and execute the LoS task). After the calibration step, we zeroed the weight of instruments on the force plate and asked the participant to stand on the balance board and perform the LoS task. The participant performed the LoS tasks with arms crossed on the chest (Fig. 3). The distance between the participant’s bare feet was equivalent to their shoulder-width. Foot position was determined on the balance board to ensure repeatability between trials. The participant performed the LoS task by receiving visual feedback on a monitor (in 150 cm away). Before collecting data, each participant learned the LoS task through training (6 repetitions). The LoS task was also performed at two other heights (levels of threat) using wooden platforms: 20 cm (low threat) and 40 cm (high threat) (Fig. 3). The height of the standing platform, in our study, implies threatening situations in everyday life (the height was equivalent to 2–3 regular stairs) [26]. To increase the threat, typically, the participant was asked to stand at the front edge (uni-directional threat) [37] or simultaneously the front and side edge of the platform (bi-directional threat) [26]. Therefore, we placed the balance board on the front and side edges of the wooden platforms (Fig. 3). To further increase the threat level, no harness system was used [26]; whereas, two spotters were present to prevent falling [26, 39]. Assessments were administered for all subjects in ascending order of the height levels [38, 57], with adequate rest between trials. At each level, the LoS task was repeated three times, and the CoP data were recorded. Also, we considered the total score of the LoS task, which ranges from zero (no target reached) to 8 (all targets were hit). Mean value of CoP parameters and the total score of LoS task were used.

### Data analysis

After removing the first 5 s of data (the time of quiet stance that was used to find home position), the signals were low-pass filtered using a 4th order Butterworth filter with a cut-off frequency of 10 Hz [67]. The filtering and the subsequent data analyses were performed by Matlab R2016b (Mathworks, MA, USA).

**Fig. 4:**
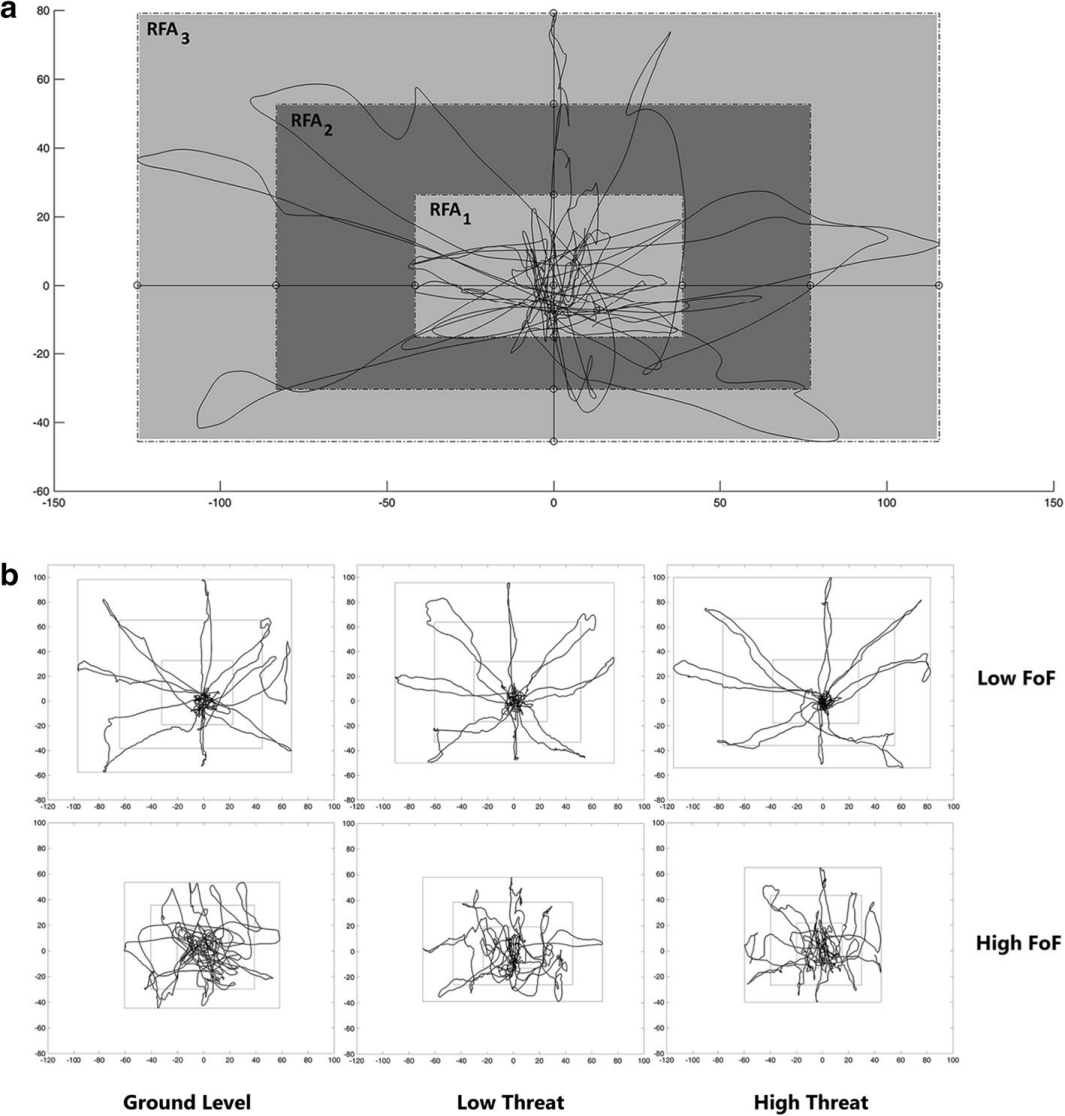
Rectangular functional areas, *RFAs*. **a** The geometrical definitions of the *RFA*s; **b** An example of CoP’s presence pattern in *RFAs* at different levels of threat (low-FoF participants in the first row and the high-FoF ones in the second row)

In this study, the focal point of the CoP analysis is the pattern of its presence in different areas. We used 75% of the CoP displacement in the calibration step to determine the location of targets relative to the home position. Eight directions were considered in the LoS task; therefore, the movement area became an octagon. For the sake of simplicity, we considered the movement area of the CoP as a rectangle. The area was then divided into three co-centric rectangles, called rectangular functional area (*RFA*_*i*_). We considered the median of the CoP data as the center of the *RFAs*. *RFAs* were built using 33%, 67%, and 100% displacement in each direction of mediolateral and anteroposterior axes (Fig. 4). The time ratio of the CoP presence in each *RFA,* calculated by Eq. (1), was named functional time ratio (*FTR*_*i*_). The subscript *i* (*i *= 1, 2, 3) indicates the corresponding *RFA*. We used *FTRs* to study the pattern of CoP presence in different areas.

1 $$FTR_{i} = \frac{{{\text{Time of the CoP presence in }}RFA_{\text{i}} {\text{while doing the LoS task }}}}{\text{Total time}} \times 100.$$

### Statistical analysis

All variables were screened for normality using the Shapiro–Wilk test. Except for the PT and the total score of LoS task, the data in all tests were normally distributed. The group differences in demographic data were checked by an independent sample *t* test. [25]. The reliability and validity of the new CoP parameters were investigated. The intra-class correlation coefficient (*ICC*_*2*, *3*_) and percentile standard error of measurement (%*SEM *= *SEM*/mean) were used to evaluate relative and absolute reliability, respectively. The *ICC* < 0.40, 0.40 ≤ *ICC* < 0.75, and the *ICC* ≥ 0.75 were interpreted as a poor, acceptable, and high relative reliability, respectively. Also, the  %*SEM* > 20%, 10% <  %*SEM* ≤ 20%, and  %*SEM* ≤ 10% were interpreted as a poor, acceptable, and high absolute reliability, respectively. Pearson’s correlation coefficient of determination (*R*^2^) was used to estimate the correlation between the normally distributed variables. Spearman correlation coefficient was used to investigate the correlation between PT and CoP parameters. The correlation values of 0.00–0.19, 0.20–0.34, 0.35–0.50, and > 0.50 were interpreted as negligible/not, weak, moderate, and strongly correlated, respectively [68]. The repeated-measure ANOVA with Bonferroni post hoc tests was used to show the differences in the CoP parameters at three levels of height in each group; also, Using Friedman and Dunn–Bonferroni post hoc tests, the differences in the total score of LoS task at three levels of height was investigated. The binary logistic regression was used to predict the odds of a participant being in the low or high-FoF groups based on the CoP parameters. Among the predictive parameters, we considered a correlation higher than 0.80 as multi-collinearity [69]. The Receiver Operating Characteristic (ROC) curve was used to investigate the power of the CoP parameters in discriminating low-FoF from high-FoF participants. Overall, in the analysis of ROC curves, the larger area under the curve (*AUC*) represents a suitable model. The Corrected Akaike’s Information Criterion (*CAIC*) was calculated to estimate the quality of each model, relative to other models. The model with the lowest *CAIC* was chosen as the best-fitting model in predicting the intensity of FoF [70]. The Youden Index was also used to determine optimal cutoff value [71]. Also, models were trained and tested using fivefold cross-validation [72]. We reported the classification performance using error estimation, sensitivity, and specificity metrics. The statistical significance threshold was set at *p* ≤ 0.05 and the confidence intervals (*CI*) for *ICC* was 95%. Statistical analyses were performed using the IBM SPSS Statistics version 22.0 (NY, USA).

## Data Availability

The data analyzed during the current study are available from the corresponding author on reasonable request.

## Abbreviations

AUC: Area Under the Curve
BBS: Berg Balance Scale
CAIC: Corrected Akaike’s Information Criterion
CI: Confidence Interval
CNS: Central Nervous System
CoP: Center of Pressure
FES-I: Falls Efficacy Scale-International
FoF: Fear of Falling
FTR: Functional Time Ratio
HADS: Hospital Anxiety and Depression Scale
HY: Hoehn and Yahr Scale
ICC: Intraclass Correlation Coefficients
LoS: Limit of Stability
MoCA: Montreal Cognitive Assessment
PD: Parkinson’s Disease
PT: Pull Test
RFA: Rectangular Functional Area
ROC: Receiver Operating Characteristic
SEM: Standard Error of Measurement
TUG: Timed Up and Go

## Footnotes

1 *N* = 10 *k*/*p*; where *p* is the smallest of the proportions of negative or positive cases in the population, and *k* is the number of independent variables. Since 70% of PD patients reported activity limitations due to FoF, we considered the proportion of negative cases in the population, 0.30 (30%). Also, in this study, we have one covariate to include in the model; N = 10 × 1/0.30 ≈ 33.
